# Supplementary material for: Accounting for differences between Infinium MethylationEPIC v2 and v1 in DNA methylation–based tools
Source: Life Sci Alliance. 2025 Jul 8;8(9):e202403155. doi: 10.26508/lsa.202403155 (PMC12238762; doi:10.26508/lsa.202403155)
Supplement: Supplementary file 2 [file LSA-2024-03155_TableS2.docx]

### Table of contents

**Supplementary Table S1.** Summary of within and between EPICv1 and EPICv2 technical replicates Spearman correlation, RMSE, and ICC.

**Supplementary Table S2.** Summary of spearman correlations, RMSE between EPICv1 and EPICv2 on array and probe level, and pooled standard deviation on probe levels (721,378 shared probes)

**Supplementary Table S3.** Summary of spearman correlations between EPICv1 and EPICv2 and pooled standard deviation on probe level.

**Supplementary Table S4.** Summary of probes with spearman correlations ≤ 0.7 between EPICv1 and EPICv2 and pooled standard deviation over the first quartile on probe level using different levels of preprocessing

**Supplementary Table S5.** Probes shared among 450K, EPICv1 and EPICv2, and with Spearman correlation ≤ 0.7 between EPICv1 and EPICv2 in the four cohorts and overlap with previously published references comparing 450K and EPICv1.

**Supplementary Table S6.** Summary of probe counts using various Spearman correlations and pooled standard deviation thresholds.

**Supplementary Table S7.** Summary of spearman correlations between EPICv1 and EPICv2 and pooled standard deviation on probe levels of the 2,169 probes in cell type deconvolution panels.

**Supplementary Table S8.** Commonly identified low concordance probes with Spearman rho correlation ≤ 0.70(calculated by each cohort) and pool SD more than the lower quartile pooled SD (calculated by each cohort), commonly across VHAS, CLHNS, CALERIE, BeCOME

**Supplementary Table S9.** Overlap of probes based on Spearman correlation and pooled standard deviation thresholds and probes with low quality and previously published references.

**Supplementary Table S10.** Paired t-tests comparing DNA methylation-based immune cell type proportions estimated using EPICv1 and EPICv2 with IDOL probes independently for each cohort. Spearman correlation (rho), paired t-test p-values and Bonferroni adjusted p-values, and Cohen’s d effect size between EPICv1 and EPICv2 estimates obtained from matched samples in each cohort are provided.

**Supplementary Table S11.** Paired t-tests comparing DNA methylation-based immune cell type proportions estimated using EPICv1 and EPICv2 with auto selected probes, independently for each cohort. Spearman correlation (rho), paired t-test p-values and Bonferroni adjusted p-values, and Cohen’s d effect size between EPICv1 and EPICv2 estimates obtained from matched samples in each cohort are provided.

**Supplementary Table S12**. Pearson correlations of epigenetic clock estimates with chronological age in EPICv1 and EPICv2 samples of VHAS, CLHNS, CALERIE and BeCOME.

**Supplementary Table S13.** Comparison of epigenetic ages between EPICv1 and EPICv2 in first-, second-, rate-based and other epigenetic clocks.

**Supplementary Table S14.** Pearson correlation (r) and paired T tests results of EAAs of between EPICv1 and EPICv2 in Horvath pantissue, Hannum, Horvath SkinBlood, PhenoAge, and GrimAge.

**Supplementary Table S15.** Pearson correlation (r) and paired T tests results of EPICv1 and EPICv2 comparisons for DunedinPACE, DNAmTL and epiTOC.

**Supplementary Table S16.** Comparison of DNA methylation-based predictor estimations between EPv1 and EPICv2.

**Supplementary Table S17.** Pearson correlations of Garma and Quintela-Fandino’s epigenetic clock estimates with chronological age in EPICv1 and EPICv2 samples of VHAS, CLHNS, CALERIE and BeCOME.

**Supplementary Table S18.** Illumina’s 17 quality control metrics, detection p-value, beadcount, average methylated and unmethylated intensity values estimated based on EPICv1 and EPICv2 DNA methylation data. For all three cohorts, values for each metric are provided as mean (standard deviation) or percentages separated by array version.

### Supplementary Table S2. Summary of spearman correlations, RMSE between EPICv1 and EPICv2 on array and probe level, and pooled standard deviation on probe levels (721,378 shared probes)

| **Array level** | | | | |
| --- | --- | --- | --- | --- |
|  | **VHAS** | **CLHNS** | **CALERIE** | **BeCOME** |
| Mean array level Spearman rho (range;sd) | 0.9761(0.9688-0.9803; sd: 0.003) | 0.9759(0.9729-0.9792; sd: 0.002) | 0.976(0.9682-0.9807; sd: 0.0029) | 0.9737(0.9684-0.9766; sd: 0.0028) |
| Mean array level RMSE | 0.0384(0.0362-0.0421; sd: 0.0014) | 0.0401(0.0375-0.0434; sd: 0.0015) | 0.0362(0.0336-0.0436; sd: 0.0023) | 0.0463(0.0417-0.0532; sd: 0.0034) |
| **Probe level** | | | | |
|  | **VHAS** | **CLHNS** | **CALERIE** | **BeCOME** |
| Mean probe level Spearman rho | 0.4109(-0.6484-0.9914; sd: 0.3483) | 0.3547(-0.9071-1; sd: 0.3878) | 0.3863(-0.7443-0.9957; sd: 0.3117) | 0.2464(-1-1; sd: 0.4311) |
| Mean probe level pooled standard deviation | 0.0305(0.001-0.3751; sd: 0.0297) | 0.0235(7e-04-0.3675; sd: 0.0199) | 0.0253(9e-04-0.3586; sd: 0.0246) | 0.0261(4e-04-0.4121; sd: 0.0271) |
| Mean probe level RMSE | 0.0297(0.0013-0.9303; sd: 0.0245) | 0.0314(0.001-0.9434; sd: 0.0251) | 0.0283(0.0011-0.9296; sd: 0.0227) | 0.033(4e-04-0.9128; sd: 0.0326) |
| First lower quartile pooled SD (threshold) | 0.0101 | 0.0099 | 0.0111 | 0.0101 |

RMSE: root mean square root error; sd: standard deviation
